# Report of Cases

**Published:** 1857-08

**Authors:** D. L. M’Gugin


### Report of Cases (from page 95.)

BY PROF. D. L. M'GUGIN., A. M., M. D.

[This case was intended to follow those upon the preceding pages of the present number, but by inadvertence the copy was mislaid and its place filled by other matter before its absence was noted. With this apology to the Doctor for thus breaking the unity of his communication, we insert the case here, satisfied that our readers will be pleased to obtain the report of so interesting a case, wherever found. Eds.]

### *Hydrohpobic Hysteria.*

A case of this nature occurred in this city in the month of June, 1855, which, because of the character of the individual so afflicted, the circumstances under which it occurred and the phenomena exhibited, rendered it one of the most interesting cases which ever came under my observation, whether regarded in its moral or pathological aspect.

Mrs. C—— was a most beautiful and highly cultivated lady, and was the daughter of a French gentleman of great acquirements, of much prominence in his native country, and who was driven therefrom by one of the great revolutions which disturbed that ever-changing government, when he came to this country an exile, bringing with him this, his only child, then very young. In order to maintain himself and this child, so devoted to him, he entered one of the literary and scientific institutions, in what part of the country I am unable to say, as professor in some department of natural science. Meanwhile he was not neglectful of the education of the ardent young being for whom he seemed only to live and labor, and who grew up an accomplished scholar and a proficient in music. After she had attained to girlhood, she was overwhelmed with sorrow by the death of her only protector, and thrown upon a cold, unfeeling and uncharitable world, to struggle and buffet with poverty and dependence. Soon she undertook to instruct in music, in which she succeeded beyond even her own most sanguine expectations. She relied solely upon her own efforts, and although aid was proffered her by a generous heart and an open hand, she nevertheless preferred to struggle on, relying upon her own individual efforts. But her sprightliness, her extraordinary acquirements, her great amiability and her peculiar beauty, attracted the attention of a young gentleman, a thriving merchant of St. Louis, who married her and by whom she had one child, which died in its early childhood.

This doubtless proved too much for the integrity of her nervous system, and she became nervous and hysterical. This grew upon her, and in the summer of the year above mentioned, the husband accompanied her on a tour to St. Paul with the view to benefit her health. On her return, however, and before they reached this place, she began to complain of indisposition, attended with severe head-ache, but nevertheless reached this city, and upon their arrival sent for Dr. Hughes who found her without fever, without exalted tenderness of skin, without salivary flow and without hurried respiration. But the bowels were constipated, the urine pale and the attempt at swallowing was undertaken with some reluctance and performed with difficulty. Narcotics, antispasmodics and apperients were properly and perseveringly given, when the Dr. himself was taken down with severe indisposition, and Dr. Allen and myself were requested to see and take charge of the case. This was about two days after her arrival in this place, during which time it was manifest that she had been growing worse.

When we entered the room together, either the sudden rush of the current of air, admitted through the opened door, and falling upon her person, or the unexpected entrance of strangers, startled her and excited a slight paroxysm. What a painfully interesting spectacle here met our gaze! Her countenance was a blaze of excessive excitement; every muscle of her fine and delicate form was in a state of contractility. The bland and pleasing deportment of Dr. Allen, and his ready tact, the result of a long experience in the successful management of the insane, quieted her and she became quite calm and communicable. In myself she fancied she had found one who resembled her deceased father, by which title she continued to address me. Having secured her confidence we entered upon measures for her relief. It was very manifest that there was increased erethism of the brain and spinal marrow, the force of which would be expended in a paroxysm, when she would enjoy a lucid interval. During these short intervals her conversation, though rapid and apparently uncontrolable, was nevertheless more or less coherent, and her language was very expressive and highly chaste. She had slept very little for the last forty-eight hours notwithstanding the persevering efforts to superinduce it. She now demanded water and complained of much thirst, but when she touched it to her lips a most violent paroxysm was produced. With one hand she violently thrust the vessel from her and with the other grasping her throat, significant of a chocking spasm of the larynx and trachea, while she bounded erect in bed, uttering loud and half suppressed screams as if struggling to take breath. When first attempting to drink the appearance of the water would produce a slight shivering of dread and after some hesitation she would hurriedly, as if laboring under a feeling of desperation, seize the cup and with a sudden effort attempt to gulph it down, during which she would suspend her respiration, and then after the effort at swallowing, her conduct would resemble that of an individual who had been for a time immersed in water of low temperature. Then would follow the fierce, uncontrolable paroxysm, and the wild, expiratory screech. During these fits her countenance would be thrown into the wildest frenzy, and every motor muscle of the body under the will was thrown into the tensest state of contractility. At this stage of her disease some of the special senses were morbidly excited.

A sudden flash of lightning would excite her; the explosive sound of a gun, or a stream of cold air would produce a paroxysmal attack.

But as the case advanced, and the paroxysms became more severe, she lost much if not the entire control of her moral deportment. In order to convey, however, a correct idea of her condition when under one of those paroxysms, I will here quote the language of Dr. Allen, who, with myself, was constantly with her.

"Her moral state changed as suddenly as her physical. During an interval of excitement, her voice was subdued, her language delicate and refined, her delicacy most sensitive, her sentiments elevated, her expressions full of respectful gratitude and gentleness. But let any of the slight causes which excited her act, and at once, as by a demoniac magic, her whole nature was reversed—she became vociferously boistrous, her language coarse and vulgar, her delicacy forgotten, her expressions curses and imprecations on all around, her actions menacing and violent; the paroxysm generally vanishing gradually, the senses and affections slowly resuming their original relations, as her musical voice would utter words of penitence for the previous paroxysmal perturbation, and the scene would end in exhaustion under the utterance of a most touching, tender, and humble petition to heaven and her attendants for forgiveness and relief."

A close and particular inquiry revealed no evidence of rabid poison in this case. She had on a previous occasion suffered from similar symptoms, but in a mild form, and the absence of prominent symptoms of rabid hydrophobia, indicated the character of the enemy which was, nevertheless, equally as sure of ultimate triumph.

The pulse was now more frequent, particularly during the paroxysms, the respiration hurried and irregular, and there was also increased anxiety and restlessness. During the night the intervals grew shorter, and there was more incoherency. Her moral as well as physical condition was now more rapidly changing, and she was less easily persuaded or controled. At a still later period there was a manifest increase in the frequency of the pulse, which showed a very remarkable nervous thrill. The respiration was frequently interrupted by deep drawn sighs, the dread of water increased in intensity, and fluids of whatever kind aroused demonstrations of horror. When a short repose was enjoyed it was disturbed by a still stronger spasmodic demonstration, as if the brain during repose had acquired an increase of the erethism. Until about 2 o'clock on the following day these symptoms increased in frequency and force, when she begged to be removed to a mattrass upon the floor. After this, she sank rapidly, her breathing became slower, while her pulse declined in force but increased in frequency. Our fears were soon to be realized, by an overwhelming congestion of the brain, which took place in a short time, and she sank quietly to rest.

It was after her removal to the floor that a new feature in her case made itself manifest in the form of aphrodisiac symptoms. I have said this was a new symptom, and such was the fact, for prior to this we learned that the very opposite had been the case.

Antispasmodics, by the mouth and by enemata, of the different articles, in this class of remedies, were faithfully administered. They were also locally applied to the spine and to the neck, anteriorly, chloroform by inhalation and to the skin. In the form of inhalation it was taken with difficulty and much reluctance, and very slight effects were produced by it, owing to the impossibility on her part of inhaling it.

Such was this very remarkable and interesting case, and, although we have gone into detail in its relation, there is doubtless an omission of some important facts, as this report was written from memory.

The nosology of hydrophobia is so intimately blended with its pathology that it becomes necessary that we should show why we regarded this case as one strictly symptomatic or of the hysterical variety. Formerly it was maintained that none of the symptoms of hydrophobia ever presented themselves except when some rabid poison had been introduced. More recently, however, it was found that a spasmodic condition of the pharynx is usually attended with a great dread of water, indeed, of all kinds of fluids, where no virus had been introduced into the system. Before this fact became known this case would have been regarded as one of true hydrophobia. A better division, and a simpler one than that made by some authors, would be into *traumatic* and *symptomatic* hydropobia. In the traumatic variety a virus, peculiar to itself, is introduced into the system ; it is peculiar because it is followed by a certain class of symptoms. The phenomena exhibited indicate an erethism of that part of the brain from which spring fibres of the pharyngeal nerve near the restiform bodies, and hence the spasm.

This irritant is a poison, and the vehicle for its diffusion and distribution is the blood. It would appear also that this poison is cumulative, or in other words, organizable, and requires time for an increase of quantity to produce the usual physical effects.

The symptoms are more or less uniform in their character, if not in the period of their supervention, which shows that in order to the production of similar or identical phenomena, it is required that there be similar or identical causes. This, we admit is not always the case, but it is true with regard to poisons, as the spasms from strychnine are not such as would be produced from opium or stramonium.

May not a poison originate in the human system, somewhat analogous to that which is produced from some morbid condition of the canine species, and arising out of some degraded condition of the blood, through the mal-performance of the secretions? Is the urea retained in hysteria? else, why the pale urine? In the more exalted form which I have here attempted to describe, may not this product undergo, with other properties of the blood, still greater morbid changes, thereby producing exaggerated symptoms? That the retention of even a small amount of urea in the blood will produce cerebral symptoms, attended with mental aberation, is a fact well known.

The impossibility to trace back to an injury from the bite of any animal in many cases which have been reported, would favor the conclusion that such may often have been the case.

So few cases of *hydrophobic hysteria* have been reported, that its pathology is but little known; but in all the cases heard from, death has followed. It seems, therefore, equally fatal with the traumatic variety.

